# Supplementary material for: An Atomistic Model of a Precursor State of Light-Induced Channel Opening of Channelrhodopsin
Source: Biophys J. 2018 Aug 27;115(7):1281–91. doi: 10.1016/j.bpj.2018.08.024 (PMC6170652; doi:10.1016/j.bpj.2018.08.024)
Supplement: Document S1. Figs. S1–S7 and Tables S1 and S2 [file mmc1.pdf]

Biophysical Journal, Volume *115*

Supplemental Information

An Atomistic Model of a Precursor State of Light-Induced Channel Opening of Channelrhodopsin

Cheng Cheng, Motoshi Kamiya, Mizuki Takemoto, Ryuichiro Ishitani, Osamu Nureki, Norio Yoshida, and Shigehiko Hayashi

Table S1 Dihedral angles of the polyene chain of the chromophore (degrees). Values in parenthesis are deviations from the planarity.

	X-Ray (3UG9)	D_0	P_1	eP_2	eP_2'
C_5=C_6	-0.9 (0.9)	7.1 (7.1)	7.4 (7.4)	12.1 (12.1)	7.5 (7.5)
C_6–C_7	177.7 (2.3)	173.7 (6.3)	173.0 (7.0)	-176.9 (3.1)	175.3 (4.7)
C_7=C_8	178.2 (1.8)	-178.0 (2.0)	-174.0 (6.0)	-169.2 (10.8)	-177.1 (2.9)
C_8–C_9	-173.0 (7.0)	-176.0 (4.0)	177.7 (2.3)	-179.0 (1.0)	-179.0 (1.0)
C_9=C_{10}	179.8 (0.2)	173.6 (6.4)	-173.8 (6.2)	-171.0 (9.0)	-177.9 (2.1)
C_{10}–C_{11}	-174.0 (6.0)	-172.0 (8.0)	-179.8 (0.2)	-170.0 (10.0)	-176.0 (4.0)
C_{11}=C_{12}	179.4 (0.6)	170.4 (9.6)	-169.3 (10.7)	-169.8 (10.2)	-174.2 (5.8)
C_{12}–C_{13}	172.3 (7.7)	-178.5 (1.5)	171.6 (8.4)	-179.1 (0.9)	172.3 (7.7)
C_{13}=C_{14}	-179.6 (0.4)	167.2 (12.8)	25.8 (25.8)	6.3 (6.3)	20.6 (20.6)
C_{14}–C_{15}	-174.3 (5.7)	176.9 (3.1)	-173.4 (6.6)	-160.5 (19.5)	-166.4 (13.6)
C_{15}=N_ζ	167.1 (12.9)	176.5 (3.5)	-154.9 (25.1)	-177.1 (2.9)	-161.9 (18.1)

Table S2 Comparison of energies between the eP_2 and eP_2' states.

	E_{QM}[a]	$V_{QM\text{-}MM}$[b]
eP_2	-912.4657	-0.1354
eP_2'	-912.4588	-0.1330
	ΔE_{QM}[c]	$\Delta V_{QM\text{-}MM}$[c]
eP_2 – eP_2'	-4.3	-1.5

[a] Expectation values of the QM Hamiltonian (Hartree).

[b] Mean QM-MM interaction energies obtained at the converged cycles of QM/MM RWFE-SCF optimizations (Hartree).

[c] Difference in the energies between the eP_2 and eP_2' states (kcal/mol).

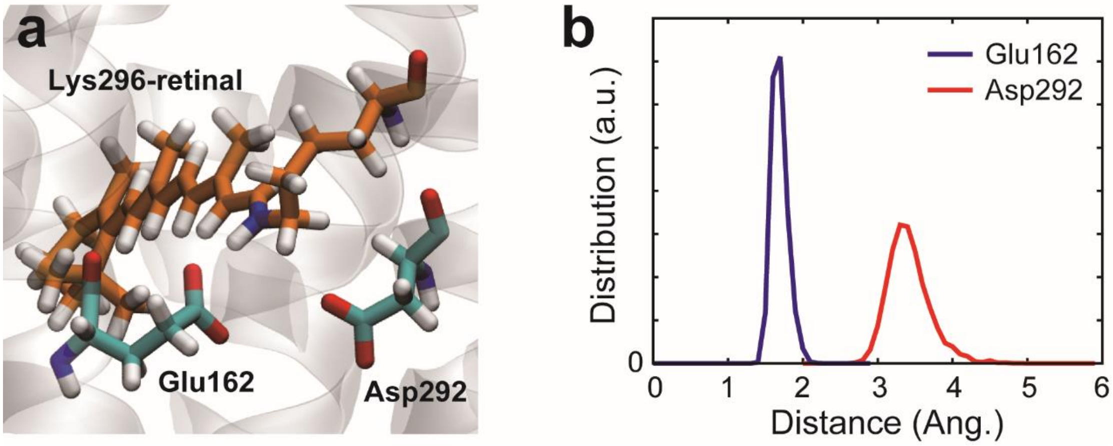

Figure S1 Salt-bridge formation of PSB with its counter ion carboxylate. a A snapshot structure around PSB from an equilibrium MD trajectory of the D_0 state. b Statistical distributions of distances between $H\zeta$ atom of PSB and the closest oxygen atoms of carboxyl groups of its counter ion carboxylates, Glu162 and Asp292, in an equilibrium MD trajectory of the D_0 state.

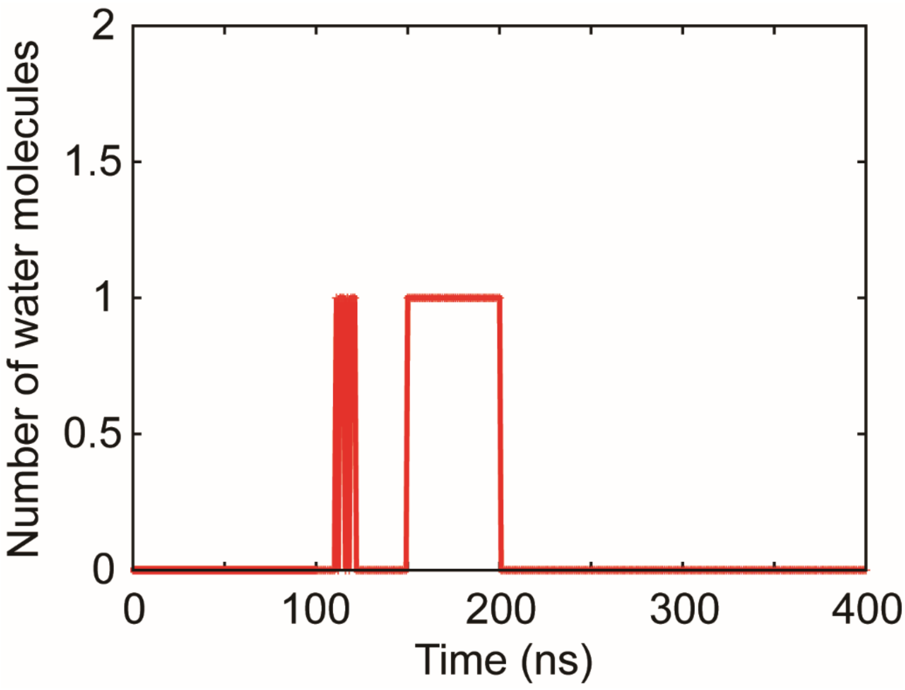

Figure S2 Transient occupation of a water molecule in a cavity at the DC-gate in an equilibrium MD simulation of the D_0 state. Water molecules of which the oxygen atoms are within 4.5 Å from the carbon atom of the carboxyl group of Asp195 were counted.

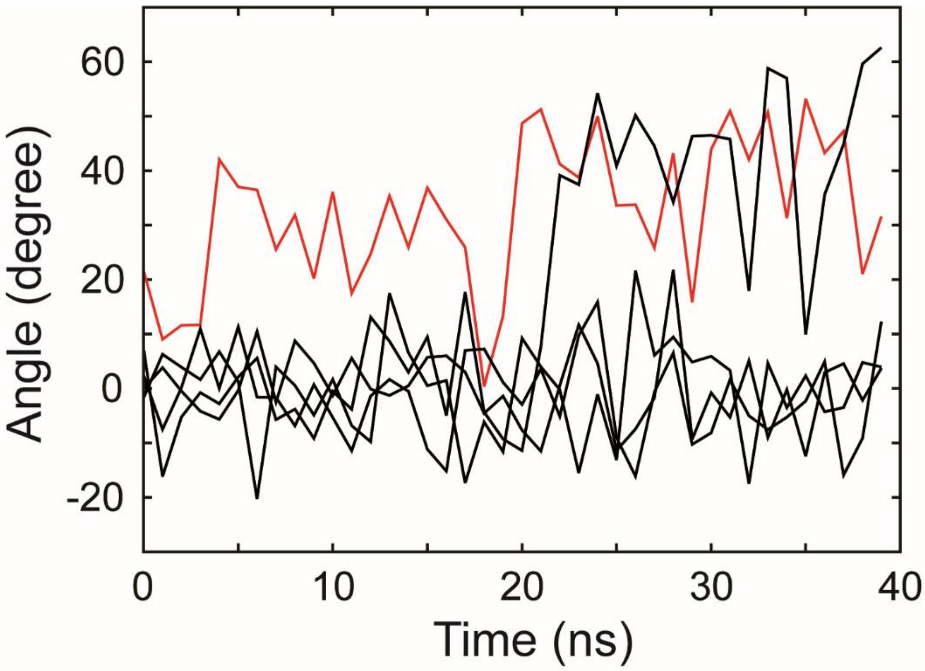

Figure S3 Time evolution of a dihedral angle of C(5-Me)-C_5-C_{13}-C(13-Me) of the chromophore during non-equilibrium MD simulations of the eP_2 state formation where C(5-Me) and C(13-Me) are the carbon atoms of methyl groups at the positions of 5 and 13, respectively. A red line indicates the time evolution of a trajectory which was followed by a QM/MM free energy geometry optimization shown in Figure S4.

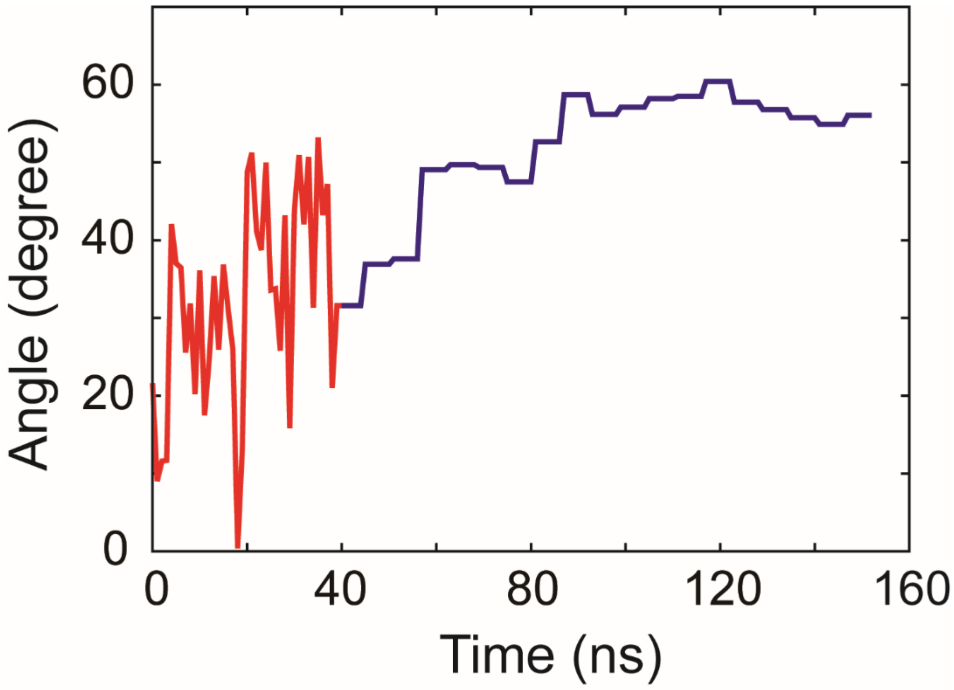

Figure S4 Time evolution of a dihedral angle of C(5-Me)-C_5-C_{13}-C(13-Me) of the chromophore during a non-equilibrium MD simulation (red) and a QM/MM free energy geometry optimization (blue) of formation of the eP_2 state.

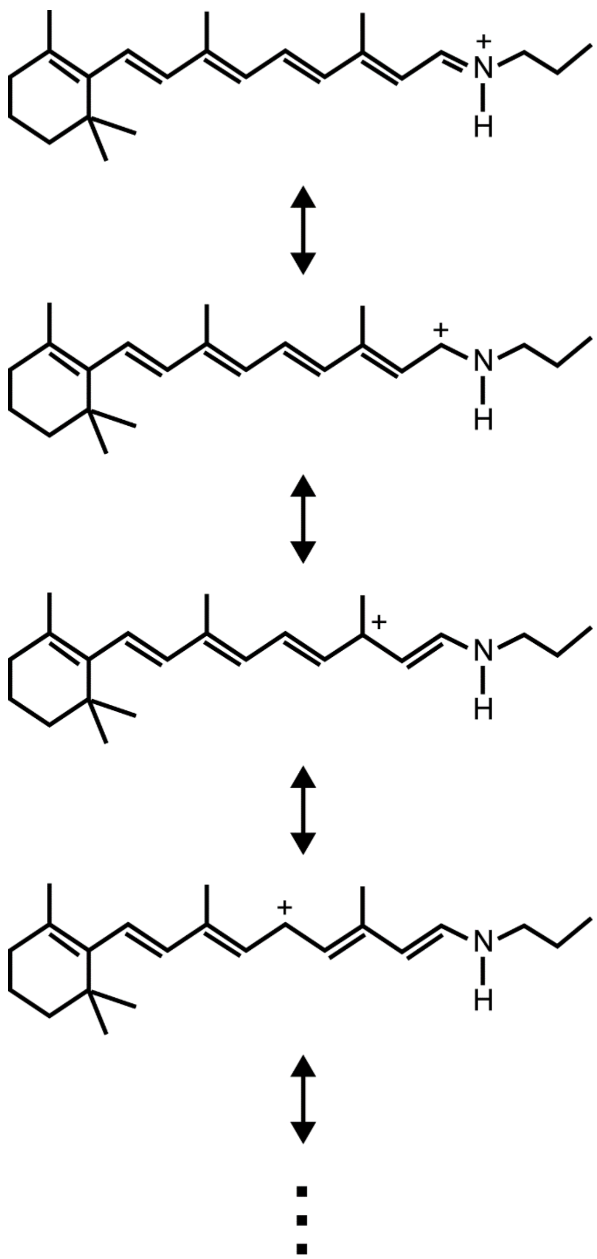

Figure S5 Resonance electronic structures of retinal protonated Schiff base. Single and double bond characters of the polyene chain is more strongly mixed through the resonance structures. Note that the resonance structures are very unstable in the deprotonated form of retinal Schiff base, and thus its single and double bond characters are more pronounced.

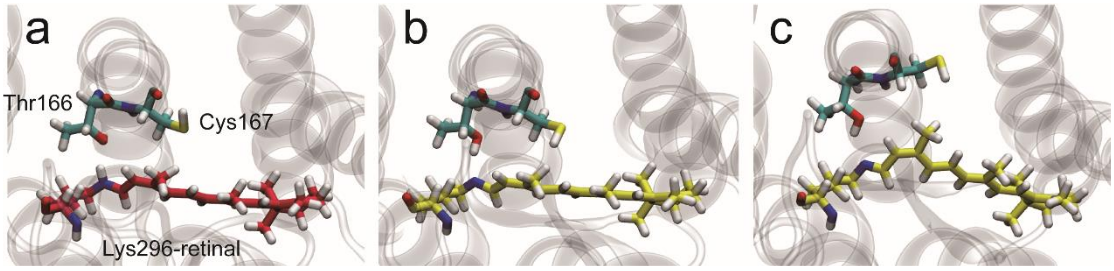

Figure S6 Free energetically minimum structures of the chromophore during formation of the eP_2 state. a-c Views of the chromophore binding site from the cytoplasmic side in the P_1 (a), eP_2' (b), and eP_2 (c) states. The structures of the P_1 and eP_2 states are the same as those shown in Figure 2c,e, respectively.

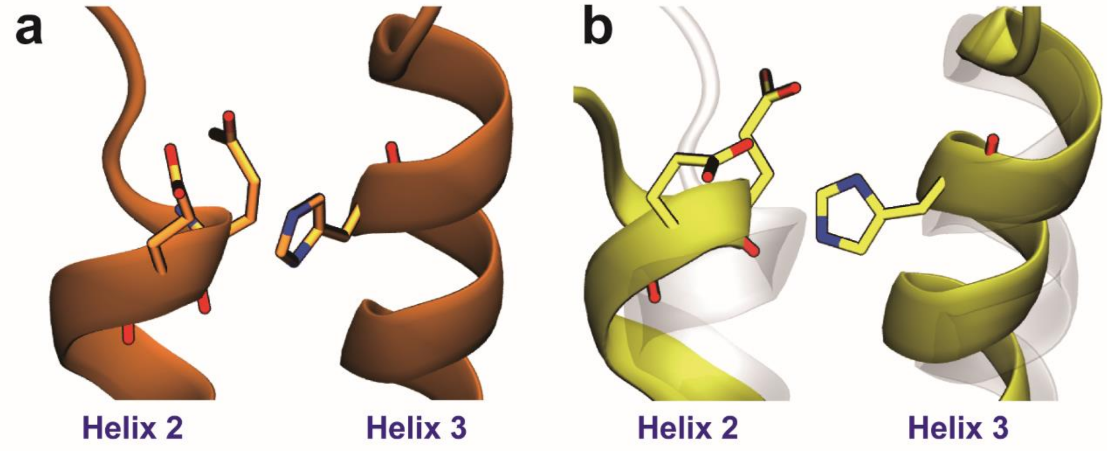

Figure S7 Conformational changes of cytoplasmic ends of Helixes 2 and 3 upon formation of the eP_2 state. a-b Snapshot structures of cytoplasmic ends of Helixes 2 and 3 in the D_0 state (a) and the eP_2 state (b), respectively, taken from equilibrium MD simulations. Side-chains of Glu121 and Glu122 in Helix 2 and that of His173 are depicted in licorice representation. The structure in the D_0 state is also drawn transparently in b.
