# Supplementary material for: An Atomistic Model of a Precursor State of Light-Induced Channel Opening of Channelrhodopsin
Source: Biophys J. 2018 Aug 27;115(7):1281–91. doi: 10.1016/j.bpj.2018.08.024 (PMC6170652; doi:10.1016/j.bpj.2018.08.024)
Supplement: Document S2. Article plus Supporting Material [file mmc2.pdf]

Biophysical Journal
Article

Biophysical Society

An Atomistic Model of a Precursor State of Light-Induced Channel Opening of Channelrhodopsin

Cheng Cheng,[1] Motoshi Kamiya,[1] Mizuki Takemoto,[2] Ryuichiro Ishitani,[2] Osamu Nureki,[2] Norio Yoshida,[3,*] and Shigehiko Hayashi[1,*]

[1]Department of Chemistry, Graduate School of Science, Kyoto University, Kyoto, Japan; [2]Department of Biological Sciences, Graduate School of Science, The University of Tokyo, Tokyo, Japan; and [3]Department of Chemistry, Graduate School of Science, Kyushu University, Fukuoka, Japan

ABSTRACT Channelrhodopsins (ChRs) are microbial light-gated ion channels with a retinal chromophore and are widely utilized in optogenetics to precisely control neuronal activity with light. Despite increasing understanding of their structures and photoactivation kinetics, the atomistic mechanism of light gating and ion conduction remains elusive. Here, we present an atomic structural model of a chimeric ChR in a precursor state of the channel opening determined by an accurate hybrid molecular simulation technique and a statistical theory of internal water distribution. The photoactivated structure features extensive tilt of the chromophore accompanied by redistribution of water molecules in its binding pocket, which is absent in previously known photoactivated structures of analogous photoreceptors, and widely agrees with structural and spectroscopic experimental evidence of ChRs. The atomistic model manifests a photoactivated ion-conduction pathway that is markedly different from a previously proposed one and successfully explains experimentally observed mutagenic effects on key channel properties.

INTRODUCTION

Channelrhodopsins (ChRs) are photosensitive ion channel proteins found in microbes such as green algae and have been utilized in neuroscience as optogenetic tools (1). When ChRs are heterologously expressed in a neuron, its neural activity can be controlled by illuminating light, which induces cell-specific change of a membrane potential through the photosensitive ion conduction. This optogenetic approach thus enables precise neuronal control for microscopic understanding of neuronal activities in psychiatric states (2) and a therapy for restoring vision (3).

Atomistic views of the light-sensitive ion channels of ChRs have been provided by x-ray crystallography, which determined an atomic structure of a chimeric form of channelrhodopsin-1 (ChR1) and channelrhodopsin-2 (ChR2) called C1C2 (4) (Fig. 1 *a*) and that of ChR2 itself (5). ChR forms a homodimer, and each monomer consists of seven transmembrane helixes constituting a cation channel. At the middle of the channel, a chromophore molecule responsible for reception of light, retinal, is covalently bound to a lysine of the protein through a protonated Schiff base (PSB) linkage.

The channel functions of ChRs have been investigated through biochemical, spectroscopic, and computational approaches (6–8) since the discoveries of ChR1 (9) and ChR2 (10). The light-induced ion channel conduction appears in a photocycle triggered by photoabsorption of the retinal protonated Schiff base (RPSB) chromophore (Fig. 1 *b*). In the resting dark state in which the ion channel is closed, the RPSB chromophore in the all-*trans* conformation resides in the binding pocket. Upon photoabsorption, the chromophore molecule undergoes ultrafast isomerization from the all-*trans* conformation to the 13-*cis* one, which completes within a few picoseconds. The primary photochemical reaction then induces conformational changes of the chromophore and the protein accompanied by changes of protonation states of titratable groups in the channel, which lead to formation of the open state of the channel. The photoactivation mechanism is analogous to that of a light-driven proton pump of a representative microbial rhodopsin, bacteriorhodopsin (bR), which has been studied for more than four decades since its discovery. In fact, ChR2 was characterized as a leaky proton pump (11).

However, the temporally transient nature of the photoinduced ion channel conduction of ChRs has hampered

Submitted June 26, 2018, and accepted for publication August 13, 2018.

*Correspondence: noriwo@chem.kyushu-univ.jp or hayashig@kuchem.kyoto-u.ac.jp

Editor: Alan Grossfield.

https://doi.org/10.1016/j.bpj.2018.08.024

© 2018 Biophysical Society.

This is an open access article under the CC BY license (http://creativecommons.org/licenses/by/4.0/).

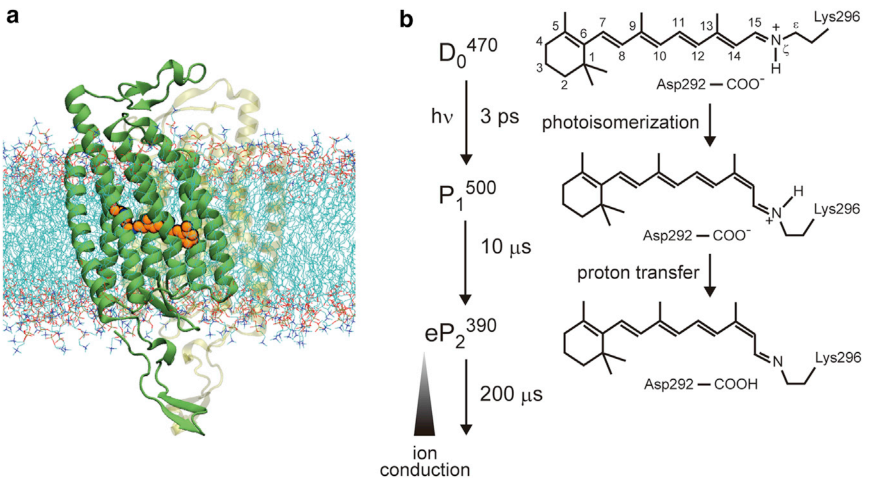

FIGURE 1 Protein structure and photoactivation scheme of ChR. (*a*) A simulation system. A dimeric form of C1C2 is embedded in a lipid bilayer. One of the monomers is shown in a transparent representation for clarity. A retinal chromophore is drawn in orange. (*b*) A scheme of the photoactivated channel opening. In the dark state (D_0^{470}), the retinal chromophore in the all-*trans* conformation resides in the protein. The photoisomerization immediately leads to the P_1^{500} intermediate, in which the conformation of the chromophore is changed to the 13-*cis* form. A proton transfer from the Schiff base to its counterion carboxylate of Asp292 with a time constant of 10 μs then generates the early stage of P_2^{390} state (eP_2^{390}), in which the ion conduction is not yet started. The ion conduction gradually develops after the formation of eP_2^{390} with a time constant of 200 μs. To see this figure in color, go online.

detailed microscopic examination of the channel conduction. Lack of clear understanding of the ion-conducting open state and its photoactivation mechanism has been a major obstacle to improvement of functional properties of ChRs unfavorable for optogenetics such as small ion conductance (below 1 pS single-channel conductance (10,11)) and poor cation selectivity.

Recent Fourier transform infrared (FTIR) vibrational spectroscopic experiments and molecular dynamics (MD) simulations have provided fruitful insights into the molecular mechanism of the photoactivation of the ion channels (12–19). Nevertheless, the molecular mechanism of the photosensitive opening of the ion channels is still under debate. A critical issue is the role of protonation change of a well-conserved glutamate, Glu90 in ChR2 and Glu129 in C1C2 (Fig. 2 *a*), which was found to constitute a constriction site in a putative channel in the x-ray crystallographic structures (4,5) in the photosensitive channel opening. Some FTIR measurements observed that the protonated Glu90 in ChR2 in the dark state underwent deprotonation upon the formation of the photoactivated state (13,16). As shown by MD simulations (12,13,18), introduction of a negative charge by the deprotonation of the glutamate was proposed to open the constriction site and form a pore developing throughout the channel accompanied by movements of the transmembrane helixes. The helix movements seen in the MD simulations are consistent with experimental observations of structural analyses (20–22).

However, other FTIR measurements found different behaviors of the protonation states of the glutamates in the photoactivations. Lórenz-Fonfría et al. observed that Glu90 in ChR2 was kept protonated in the photoactivated channel opening state and underwent deprotonation only in a desensitized state (14). Inaguma et al. also showed that in the photoactivated state, Glu129 in C1C2 was protonated and its hydrogen-bond was unchanged (16). The observations are in line with the experimental evidence that mutations of the glutamate at the constriction site with nontitratable alanine or glutamine residues reduced the photoinduced ion current by only half and does not completely abolish it (12,18), indicating the noncritical role of the protonation state of the glutamate at the constriction site in the channel opening. Lórenz-Fonfría et al. instead proposed that

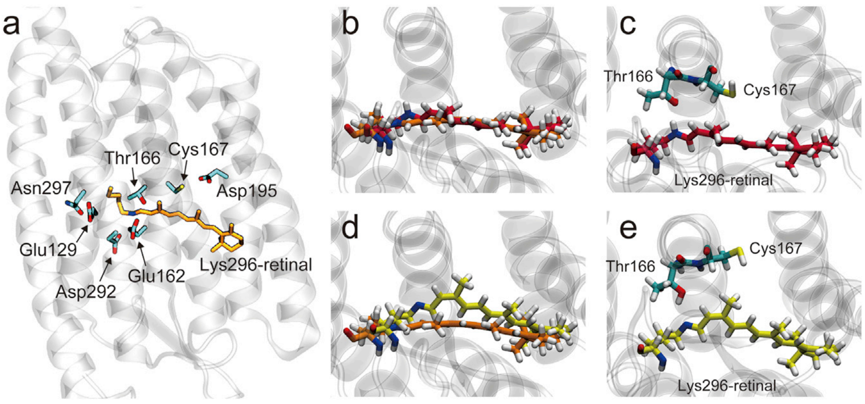

FIGURE 2 Molecular structures of the retinal chromophore binding site in C1C2. (*a*) Functionally important residues around the chromophore in the D_0 state. (*b* and *c*) The structure of the chromophore in the P_1 state drawn in red, superimposed on that in the D_0 state in orange (*b*) and with nearby residues in helix 3, Thr166 and Cys167 (*c*). Views are from the cytoplasmic side. (*d* and *e*) The structure of the chromophore in the eP_2 state drawn in yellow, superimposed on that in the D_0 state in orange (*d*) and with the nearby residues in helix 3 (*e*). To see this figure in color, go online.

hydration of the transmembrane helixes kinetically governs the channel opening (15).

The atomistic mechanism of the light-sensitive ion transports of microbial rhodopsins has widely been investigated through molecular simulation approaches (12,13,17–19,23–29). In the case of bR, molecular simulation studies (24,25) have successfully captured mechanistic features of conformational changes of RPSB and translocations of water molecules in the early stages of the photoactivation, which are in line with recent experimental observations of serial femtosecond x-ray crystallography (30).

Despite the success for the early stage of the photoactivation, however, MD simulations of the photoactivation processes on longer timescales suffer from difficulty in accurately describing conformational changes of the RPSB chromophore possessing complex electronic structure and the surrounding proteins undergoing large conformational changes. Because the inaccurate description could introduce erroneous behavior of the molecular dynamics more in a longer trajectory, the inaccuracy needs to be minimized in an MD simulation on a longer timescale.

The difficulty described above has partly been overcome by the quantum mechanical/molecular mechanical (QM/MM) reweighting free energy-self consistent field (RWFE-SCF) method, which is a free-energy geometry optimization technique developed recently (31,32). The QM/MM RWFE-SCF method is designed to be completely variational and computationally highly efficient, allowing one to optimize the electronic wave function and the molecular geometry of the QM molecule at the ab initio level of theory on a free-energy surface constructed with a statistically extensive conformational ensemble of the MM protein environment obtained by long-time MD simulations. The QM/MM RWFE-SCF method, in combination with MD simulations, was applied to the photoactivation of a visual receptor rhodopsin and successfully obtained atomistic structures of photointermediates formed on the submicrosecond timescale, of which the electronic and vibrational spectroscopic properties agree with experimental ones (33). The method was also utilized to theoretically design color variants of the microbial rhodopsin optogenetics tools involving significant conformational changes of the chromophore, which were confirmed by spectroscopic and x-ray crystallographic measurements (34).

Here, we report a theoretical study on the photoactivation of C1C2 by means of the molecular simulations. We focus on the first two intermediate states of the photocycle, P_1 and the early state of P_2 (eP_2) (Fig. 1 *b*). The P_1 state is the first intermediate formed from the dark state (D_0) within several picoseconds immediately after the photoisomerization of the chromophore. An experiment showed that the following eP_2 state generates with a time constant of 10 μs and is accompanied by a proton transfer from PSB to a counterion carboxylate of Asp292 (14) (Fig. 2 *a*). It was also experimentally shown that, upon the formation of eP_2, partial hydration of the transmembrane helixes takes place, although the ion conduction is not established yet (15). The eP_2 state is therefore considered to be a precursory signature state of the ion-conducting open state, which forms on a submillisecond timescale.

Unlike the previous simulation studies (12,13,18), we kept the protonation state of Glu129 at the constriction site protonated based on the experimental evidence (16) and thus examined a novel, to our knowledge, molecular mechanism of the photoinduced channel opening without the deprotonation of the glutamate at the constriction site. Through accurate structural refinements of the intermediate states by the QM/MM RWFE-SCF geometry optimization with an ab initio density functional theory (DFT) description and analysis of hydration of the transmembrane helixes by a statistical integral equation theory—the three-dimensional reference interaction site model (3D-RISM) theory (35–38), which can capture statistically converged water distribution even deep inside the protein—we determined a plausible model of the eP_2 state that agrees with experimental observations of protein structural changes (20–22) and helix hydration (15). The eP_2 structure clearly shows precursory formation of an ion channel that is different from the previously proposed ones (12,13,18,19) and successfully explains experimental evidence on the noncriticality of the deprotonation of the glutamate at the constriction site for the channel opening. This model also successfully sheds light on molecular mechanism of a key functional moiety controlling the channel opening called the DC gate (39), which is not evident in the previously proposed models. This study provides an atomistic model of the photoinduced ion channel that opens the way for microscopic understanding of the channel properties and their improvement through rational design.

METHODS

A molecular simulation model constructed previously (18) was employed. An x-ray crystallographic structure of a dimeric C1C2 (4) (Protein Data Bank (PDB): 3UG9) was embedded in a 1-palmitoyl-2-oleoyl-sn-glycero-3-phosphocholine lipid bilayer and water solvent with Na^+ and Cl^- ions of 0.15 M in a periodic boundary condition (Fig. 1 *a*). The total number of atoms was 194,121. The protonation states of titratable groups were assigned to the standard protonation states at neutral pH by PROPKA (40), except for Glu129, Glu136, and Asp195, which were protonated based on the experimental observations (14,16). Glu122 was deprotonated following experimental evidence (34).

Structural refinements were carried out by QM/MM RWFE-SCF calculations (31,32,41), which macroiterate classical MD conformational sampling of the MM region and QM/MM geometry optimization of the QM region on a free-energy surface constructed with the MM conformational samples. The MD simulations and the QM/MM geometry optimizations were performed with program packages NAMD (42) and GAMESS (43) with the QM/MM interface implemented, respectively.

The MM interactions were described with the CHARMM27 force field (44) for the protein, CHARMM36 for the lipid (45), previously developed force fields for the protonated and deprotonated states of

the retinal chromophore (18,46), and the TIP3P model for water molecules (47). The short-range interactions were cut off at a distance of 12 Å with a force-switching function beginning at 10 Å (48), and the long-range electrostatic interaction was treated with the particle mesh Ewald method (32,49). In each macroiteration of QM/MM RWFE-SCF calculations, 20,000 MM conformations were sampled from the last 4 ns of the MD trajectory for 6 ns obtained by the classical MD simulation in the isothermal-isovolumetric (NVT) condition with a fixed QM geometry.

The QM region consists of the retinal chromophore in a monomer (chain A) of the dimeric form. A link hydrogen atom was added at the QM-MM boundary. The free energy geometry optimizations of the QM region were performed at the DFT/M06-2X/6-31G* level of theory. The number of basis functions was 500 for D_0 and P_1 and 495 for eP_2. Restrained parameters for restrained electrostatic potential charge operators utilized in QM/MM RWFE-SCF calculations were set to 4×10^{-2} for the boundary atoms and 4×10^{-3} for the rest of the QM atoms. The convergence criterion of the geometry optimization was set to 5.0×10^{-4} Hartree/Bohr for the largest component of the gradient of the QM region.

Initial structures of the D_0 dark state and the P_1 and eP_2 intermediate ones for the structural refinements by QM/MM RWFE-SCF calculations were obtained by classical MD simulations. The last coordinates and velocities of an equilibrium MD simulation of the D_0 state for 100 ns were chosen for the QM/MM RWFE-SCF geometry optimization of the D_0 state. The same coordinates and velocities were also employed for the isomerization simulation to produce the P_1 state. In the simulation of the isomerization to produce the P_1 state, the potential periodicity of the dihedral angle around the $C_{13}{=}C_{14}$ bond was switched from a double-well potential having minima in both *cis* and *trans* conformations to a single-well potential having a minimum only for the *cis* one (11). After an equilibrium simulation in the C_{13}-*cis* conformation for 500 ps, the QM/MM RWFE-SCF free-energy geometry optimization of the P_1 state was carried out.

The free energetically optimized structure of the P_1 state was employed as the initial structure for the simulation of the eP_2 state formation. The protonation states of the Schiff base of the chromophore and its counterion carboxylate of Asp292 were switched to be deprotonated and protonated, respectively (14). For the simulation of the eP_2 state formation, three sequential equilibrations for 10 ns each with fixation of heavy atoms of the protein and the chromophore, constraint of them with harmonic potentials with the force constant of 0.5 kcal/mol • $Å^2$, and constraints of heavy atoms of the chromophore and Asp292 with the same harmonic potentials were first performed. A following equilibrium MD simulation with the constraints of the chromophore for 40 ns was then carried out. Five nonequilibrium simulations without constraints were started from initial coordinates and velocities taken at every 10 ns of the last equilibrium MD trajectory. The last structure of the first nonequilibrium trajectory was employed for the QM/MM RWFE-SCF geometry optimization of the eP_2 state (Figs. S3 and S4).

After the QM/MM RWFE-SCF optimizations of the D_0, P_1, and eP_2 states, equilibrium MD simulations of those states for 400 ns each in which the QM geometries were fixed at the optimized ones were performed for structural analysis. Distributions of water molecules in the protein were also examined by an integral equation theory of molecular liquids, 3D-RISM (35–37). We employed the Kovalenko-Hirata closure equation coupled with the 3D-RISM equation (38). In the 3D-RISM calculations of water distributions, all the explicit solvent water molecules and ions were stripped from the sample structures obtained by the MD simulation for 400 ns. The solvation structures were calculated for 4000 conformational samples extracted every 100 ps from the MD trajectory of each state. In addition, all the lipid molecules except for 100 lipids near the protein were removed. The same potential parameters as in the MD simulation were used. The temperature was 300 K, and NaCl solution (0.15 M) was assumed. All the 3D-RISM calculations were conducted using the in-house 3D-RISM codes developed by Maruyama et al. (50,51). Molecular images are created with VMD (52).

RESULTS

D_0 state

The free energetically optimized geometry was determined by a QM/MM RWFE-SCF calculation (see Methods). The number of the macroiterations was 30, and thus the optimized structure was obtained with the MD trajectory for 180 ns. The overall geometry of the polyene moiety of the RPSB chromophore in the all-*trans* form was optimized to be almost planar, as seen in the x-ray crystallographic structures (4,5) (Table S1). However, the positively charged PSB part is slightly twisted toward Glu162, which is one of the two counterion carboxylates (Glu162 and Asp292) positioning almost symmetrically to PSB (Figs. 2 *a* and S1) because of a tight salt-bridge formation with it, which persisted in the equilibrium trajectory of the optimized structure for 400 ns (Fig. S1). The asymmetric salt bridge between PSB and Glu162 is consistent with an experimental evidence of an FTIR measurement (53). As a result, the dihedral angle of the isomerizing bond, $C_{13}{=}C_{14}$, remarkably deviates from the planarity by 12.8° (Table S1).

A recently determined x-ray crystallographic structure of ChR2 showed that a water molecule occupies a cavity at the DC gate and forms hydrogen bonds with Cys128 and Asp156, respectively (5), which is not clearly observed in an x-ray crystallographic structure of C1C2 (4). The occupation of the water molecule at the DC gate in ChR2 was also suggested by a computational study (17) in which the occupation was maintained in an MD trajectory for 100 ns. In this longer MD trajectory for 400 ns at the free energetically optimized geometry, water molecules penetrated into the DC gate from the bulk water environment through the protein-membrane interface and transiently stayed around the DC gate for 20 and 60 ns, respectively (Fig. S2). The transient occupations indicate that the cavity at the DC gate is accessible to water molecules even though it is located deep inside the protein, whereas the interaction of a water molecule with the cavity is not strong enough to bind the water molecule for a long time. The weak occupation is consistent with the x-ray crystallographic model of C1C2 (4).

P_1 state

Because of the pretwisted conformation at $C_{13}{=}C_{14}$ described above in the D_0 state, the PSB part of the chromophore in the photoisomerization simulated by an MD simulation (see Methods) rotated clockwise around the axis from the PSB side to the β-ionone ring one. Because of the short time nature of the P_1 state formation, the major

conformational changes were localized in the PSB part of the chromophore and the β-ionone ring half of the chromophore from the isomerization bond underwent very few conformational changes. The locally distorted geometry in the 13-*cis* form also persisted in the following QM/MM RWFE-SCF free-energy geometry optimization with an MD sampling trajectory for 150 ns (Fig. 2, *b* and *c*). The localization of the conformational changes in the PSB part in the early intermediate was also observed in bR (24,27,54). Deviations from the planarity around $C_{15}{=}N_\zeta$ and $C_{13}{=}C_{14}$ in the free energetically optimized structure are ~25° (Table S1). Consequently, PSB is highly twisted and is hydrogen bonded to the hydroxyl group of Thr166. Such a hydrogen-bond formation of PSB was also found in a structural model of the KL intermediate state of bR in a previous simulation (24), in which PSB is hydrogen bonded to the hydroxyl groups of Thr89 corresponding to Thr166 of C1C2.

Early P_2 state

Nonequilibrium MD simulations of the eP_2 state starting from the free energetically optimized conformation of the chromophore in the P_1 state (see Methods) and a following free-energy geometry optimization by QM/MM RWFE-SCF method showed a significant tilt of the central part of the polyene chain of the chromophore (Fig. 2, *d* and *e*). In two trajectories out of the five independent nonequilibrium MD simulations, time evolution of a dihedral angle between the C-C bonds of the 13-methyl group and the 5-methyl one, which represents the tilting of the 13-methyl group originating from the extensive torsion of the polyene chain in the β-ionone ring half, exhibited a wide fluctuation in 10–50° (Fig. S3), indicating high flexibility of the tilting conformational change of the chromophore in the binding pocket. Then, from the last conformation of one of the two trajectories showing the wide fluctuation, a free-energy geometry optimization by the QM/MM RWFE-SCF method was carried out (Fig. S4). Significant development of the dihedral angle from 32 to 56° was observed in the following geometry refinement of the polyene conformation by the highly accurate ab initio quantum chemistry description with an MD sampling trajectory for 126 ns (Fig. S4).

Unlike the P_1 state, extensive torsion in the β-ionone ring half of the polyene chain arises (Table S1). Consequently, the 13-methyl group of the chromophore pushes the side chain of Cys167, and helix 3 moves outward around Cys167 (Fig. 2, *c* and *e*). In contrast, the local and strong distortion in the Schiff base region of the chromophore in the P_1 state is largely reduced in the formation of the eP_2 state with the deprotonated Schiff base (DSB) (Table S1), counterbalancing the extensive torsion in the β-ionone ring half. The double-bond electronic character of $C_{13}{=}C_{14}$ and $C_{15}{=}N_\zeta$, which are strongly twisted by ~25° in the P_1 state as described above, becomes large upon the deprotonation of the Schiff base in the formation of the eP_2 state because of loss of resonance structures of the positive charge in its protonated form (Fig. S5). The increase of the double-bond character is consistent with the significant reduction of the torsions around the $C_{13}{=}C_{14}$ and $C_{15}{=}N_\zeta$ bonds in the eP_2 state with DSB, i.e., deviations from the planarity are 6 and 3°, respectively (Table S1). The arrested torsion of DSB preserves its hydrogen bond with the hydroxyl group of Thr166—although DSB becomes the hydrogen-bond acceptor (Fig. 2 *e*)—and induces the counterextensive torsion in the β-ionone ring half of the polyene chain.

The nonequilibrium MD simulations of the eP_2 state showed that the chromophore conformation without the tilting similar to the P_1 one was also maintained in several trajectories (Fig. S3). However, relative stability of those two conformations was not accurately determined by the nonequilibrium MD simulations because of a poor description of the classical MM force field of the conjugated chromophore employed. Thus, to quantitatively confirm the energetics associated with the tilting conformational change of the chromophore, we also free energetically optimized the chromophore with DSB in the absence of the tilting. A free-energy minimum without the tilting (the eP_2' state) was obtained by a QM/MM RWFE-SCF geometry optimization with an MD sampling trajectory for 78 ns from the initial structure of the nonequilibrium MD trajectory, i.e., the structure at $t = 0$ in Fig. S4, in which the chromophore conformation was constrained at that in the P_1 state. The optimized structure of the chromophore in the eP_2' state stayed close to that in the P_1 one (Fig. S6; Table S1), i.e., the double bonds in the DSB region are highly twisted with slight relaxation and the extensive torsion in the β-ionone ring half is absent. As discussed above, the expectation value of the QM Hamiltonian, which mainly represents the conformational energy of the chromophore, in the eP_2' state is significantly larger by 4.3 kcal/mol than that in the eP_2 state because of the stronger torsions of the double bonds in the DSB region (Table S2). Moreover, the mean QM-MM interaction energy in the eP_2' state at the converged cycle of the QM/MM RWFE-SCF optimization is also higher by 1.5 kcal/mol than that in the eP_2 state. The eP_2 state is therefore likely to be energetically more stable than the eP_2' state, although the definitive determination of the relative stability requires a more elaborated and computationally highly demanding free-energy calculation (32,34,41), which is now ongoing in our laboratory.

The conformational changes in the eP_2 state of C1C2 are markedly different from those in the corresponding state of bR, i.e., the early M state, in which the extended tilt of the polyene chain toward helix 3 does not take place (55). The conformational difference of the chromophore is attributed to structural differences of the binding pockets. The x-ray crystallographic structures show that the binding pocket of C1C2 is not well packed around Cys167 in helix 3 and Asp195 and Ile196 in helix 4 (Fig. 3 *a*) and produces space that can accommodate the extensively tilted polyene chain

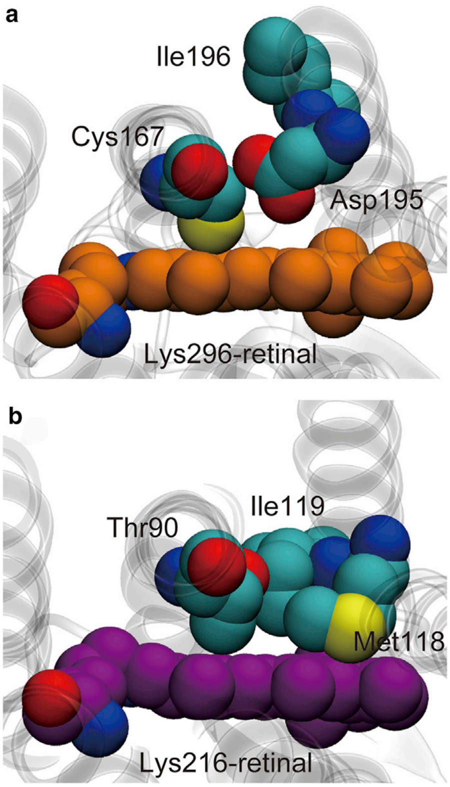

FIGURE 3 Structural comparison of the chromophore binding sites of C1C2 and bR. (*a*) The binding site of an x-ray crystallographic structure of C1C2 (PDB: 3UG9) is shown in a view from the cytoplasmic side. (*b*) The binding site of an x-ray crystallographic structure of bR (PDB: 1C3W) is shown. To see this figure in color, go online.

(4), whereas the corresponding region of bR around Thr90, Met118, and Ile119 is tightly packed (Fig. 3 *b*) and thus hinders such extensive tilt of the chain (56).

The timescale of this simulation—tens to hundreds of nanoseconds—is much shorter than the time constant of the eP_2 state formation measured experimentally, $\sim$10 μs (14). It should be noted that the timescale of the simulation does not represent the experimentally observed timescale of the eP_2 formation. Spectroscopic measurements showed that the time constant of the eP_2 state formation coincides with that of the proton translocation from PSB to its counterion glutamate (14). In this simulation, the proton transfer event was manually modeled to circumvent a computationally highly demanding task to explicitly describe the proton transfer process including formation and dissociation of chemical bonds. Thus, the experimentally observed kinetics was not dealt with in this simulation. Instead, we carried out the simulations of the conformational changes of the chromophore and the protein correlated with the proton translocation, which were also known to play crucial roles commonly in the functions of rhodopsins (6). Because the timescale of the conformational changes upon the formation of the eP_2 state observed in the simulation is much shorter than that of the proton translocation observed experimentally, it is considered that the proton translocation determines the kinetics of the eP_2 state formation as observed experimentally, and the conformational changes observed in the simulation are kinetically governed by the proton translocation event.

Channel hydration

A vibrational spectroscopic experiment for ChR2 showed that an initial helix hydration is accompanied by the formation of the eP_2 state, although the ion conduction is still inactive (15). In this simulation, remarkable redistribution of water molecules inside the protein upon the formation of the eP_2 state was observed (Fig. 4). Although the formation of the P_1 state does not significantly alter distribution of internal water molecules because the conformational change is limited in the local region of the chromophore, the extensive tilt of the polyene chain of the chromophore in the formation of the eP_2 state creates cavities inside the protein into which water molecules flow.

The 3D-RISM analysis clearly shows that new distributions of water molecules appear in the vicinity of the chromophore and His173, the latter of which is located in the putative channel in the cytoplasmic half in the eP_2 state (Fig. 4, *a* and *c*). The extensive tilt of the polyene chain including the 13-methyl group and the positional displacements of Cys167 and Ile170 in helix 3 described above (Figs. 2, *d* and *e* and 4 *d*) creates a cavity on the cytoplasmic side of the chromophore that water molecules newly occupy (Fig. 4 *c*). Furthermore, the positional shift of helix 3 induces reorientation of the side chain of His173 (His134 in ChR2) located in the same helix that exposes its main-chain carbonyl group (Figs. 4, *a–e* and S7), leading to the formation of a hydrogen bond of the carbonyl group with a water molecule, as clearly seen in a radial distribution function obtained by the 3D-RISM calculation (Fig. 4 *e*). The hydrogen-bond formation is suggested to correspond, at least partly, to the initial helix hydration observed by a vibrational spectroscopy (22).

Protein structural changes

It is experimentally known that the photoactivation of ChR2 involves significant protein structural changes (20–22). A cryo-electron microscopy observation of two-dimensional crystals of ChR2 showed displacement of helix 2, helix 6, and helix 7, the latter two of which move outward and inward, respectively (20). Furthermore, electron paramagnetic resonance studies for ChR2 revealed outward movements of the cytoplasmic sides of helix 2 and helix 6 (21,22). These simulations also showed the movement of the helixes upon the formation of the eP_2 state, consistent with the experimental observations (Fig. 5). Outward movements of helix 2 and helix 6 and inward movement of helix 7 on the cytoplasmic side were seen in the eP_2 state (Fig. 5 *b*),

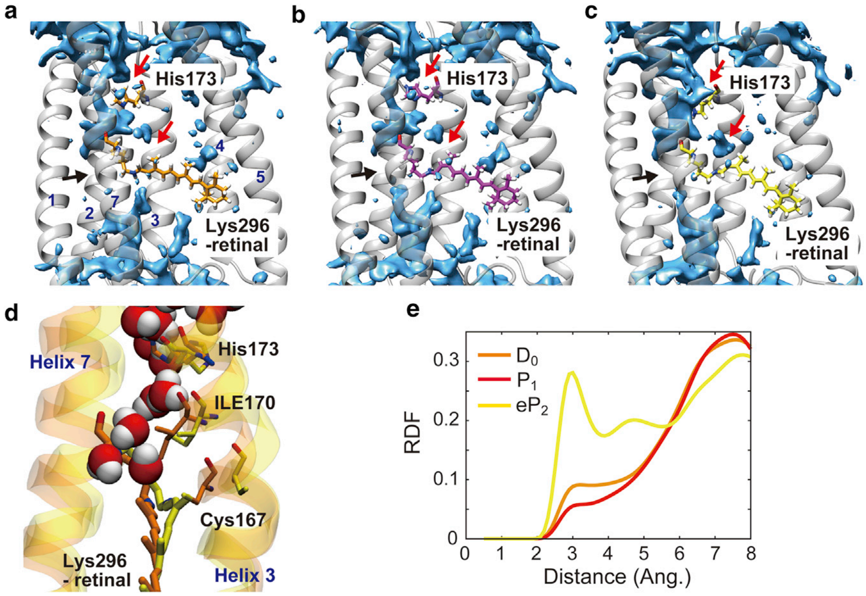

FIGURE 4 Distributions of water molecules inside C1C2. (*a–c*) Distributions of water molecules in the D_0 state (*a*), the P_1 state (*b*), and the eP_2 state (*c*) calculated by the 3D-RISM method are shown. Red arrows indicate regions where water populations newly appear upon the formation of the eP_2 state. Black arrows indicate the constriction sites. (*d*) Snapshot structures of MD simulations of the D_0 state (*orange*) and the eP_2 one (*yellow*). Water molecules in the eP_2 state are shown. (*e*) Radial distribution functions of oxygen atoms of water molecules around the oxygen atom of the main-chain carbonyl group of His173 calculated by the 3D-RISM method. To see this figure in color, go online.

whereas no significant protein structural changes were observed in the formation of the P_1 state (Fig. 5 *a*). As a result, distance between the cytoplasmic ends of helixes 2 and 6 distinctly increased by more than 1 Å (Fig. 5 *c*), representing the opening of the channel in the cytoplasmic side. The opening displacement of helix 2 is suggested to be stabilized by the reorientation of the side chain of His173 described above because of interaction of the reoriented side chain with helix 2 (Fig. S7) and the hydration of its main-chain carbonyl group (Fig. 4).

DISCUSSION

The highly accurate QM/MM free-energy optimization technique employed in our study successfully unveiled the remarkable conformation of the chromophore coupled with the large structural changes of the protein in the precursory state of the photoactivated ion conduction. Because of the complex electronic nature of the RPSB chromophore possessing the conjugated polyene structure with the resonant positive charge of PSB, an accurate molecular force field for MD simulations that is able to describe structural changes of the chromophore from a largely distorted conformation after the primary photochemical reaction is hard to obtain. QM/MM MD approaches that can handle the chromophore at the QM level of theory are also not directly applicable for the simulation of the photoactivation process involving the large protein conformational changes because of their severe limitation of simulation time.

In these simulations, the highly distorted molecular structure of the chromophore in the P_1 state was first accurately determined by the QM/MM RWFE-SCF free-energy geometry optimization with the ab initio DFT method to set a milestone toward the MD simulation of the following formation of the eP_2 state that suppresses errors due to the poor description of the force field of the chromophore mentioned above. Furthermore, the significant evolution of the tilt of the polyene chain by 24° in the following geometry refinement by the ab initio QM/MM free energy geometry optimization, with the long-time MD trajectory calculation for more than 120 ns (Fig. S4), indicates that the largely tilted conformation is the free-energy minimum in an extended configuration space when the highly complex torsional forces of the polyene chain, with the Schiff base depending on its protonation state, are accurately treated at the ab initio QM level of theory. The computed energetics

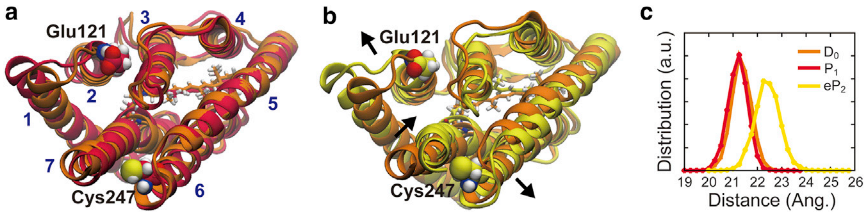

FIGURE 5 Protein structural changes in the photoactivation. (*a*) The protein structure of the P_1 state (*red*) superimposed on that of the D_0 state (*orange*) is shown in a view from the cytoplasmic side. (*b*) The protein structure of the eP_2 state (*yellow*) superimposed on that of the D_0 state (*orange*) is shown. Arrows represent movements of helix 2, 6, and 7. (*c*) Distributions of distance between the C_α atoms of Glu121 in helix 2 and Cys247 in helix 6 in equilibrium MD trajectories for 400 ns. To see this figure in color, go online.

of the eP_2 state and the eP_2' one also supports the high stability of the tilted conformation (Table S2).

Although the method can search the free-energy minimum in a very extensive free-energy basin as shown in Fig. S4, there remains a possibility that the initial geometry of the free-energy geometry optimization is not well modeled and thus the free-energy geometry optimization ends up determining a wrong structure in a different basin. To avoid this possibility, one would need to explicitly describe the photochemical process at the excited state QM levels of theory. Although we actually carried out such excited-state QM/MM simulations in previous studies for related retinal proteins (57,58), unfortunately, those simulations require highly demanding computational resources. We therefore approximated the excited-state potential surface with the simple potential function and mimicked the excited-state dynamics by the classical MD simulation (see Methods). Although the previous excited state QM/MM MD simulations revealed complex molecular dynamics in the excited state (57,58), the simulation for bR also showed that the photoproduct in the electronically ground state is well characterized by local conformational torsions in the PSB half that are consistent with this approximated MD simulation. In fact, a previous MD simulation for bR employing a procedure similar to this one (24) succeeded in properly predicting the conformational changes in the formation of the early photointermediates, which were confirmed by a recent serial femtosecond x-ray crystallography series (30). Furthermore, excited state QM/MM MEP calculations for C1C2 were also performed recently by Dokukina and Weingart (26) and Hontani et al. (28), and the conformation of the photoproduct obtained by the latter study seems consistent with this one. It is therefore considered that this approximate procedure is sufficiently reasonable to generate the proper initial guess of the free-energy geometry optimization that can search the free-energy minimum in an extensive free-energy basin.

Our study examined the photoactivation process without deprotonation of Glu129 at the constriction site, based on the experimental evidence of vibrational spectroscopy for C1C2 (16). In the previous simulation studies (12,13,18), the deprotonation of the glutamate at the constriction site was shown to induce large movement of the transmembrane helixes as seen in the experimental observations (20–22), which has been proposed to represent the opening of the channel. However, the present simulations showed that even without the deprotonation of Glu129, the significant conformational changes of the chromophore in the formation of the eP_2 state lead to the large displacement of the transmembrane helixes 2, 6, and 7, consistent with the experimental observations (20–22). The 3D-RISM analysis also identified hydrogen-bond formation of the main-chain carbonyl group of His173 in helix 3 with water molecules in the eP_2 state, which is in line with the partial hydration of the transmembrane helix observed in the FTIR experiment (14).

The atomistically determined structural model of the eP_2 state manifests to our knowledge a novel pathway of the photoactivated ion conduction that is distinctly different from the previously proposed one (Fig. 6) (12,13,18). In the previously proposed mechanism, the constriction site acts as the gate and opens when the glutamate is deprotonated in the photoactivated state, as described above. In our structural model without the deprotonation of the glutamate, on the other hand, the significant tilt of the polyene chain creates a cavity on the cytoplasmic side of the binding pocket, which is filled with water molecules and almost connects to the channel in the cytoplasmic half (Figs. 4, *c* and *d* and 6). It is noteworthy that the cytoplasmic cavity in the vicinity of the binding pocket is more pronounced in the x-ray crystallographic structure of ChR2 determined recently (5). Moreover, the tilt of the 13-*cis* polyene chain in the bent form is expected to create a cavity in its concave side (Figs. 2 *e* and 6), which can connect the newly formed cytoplasmic cavity and the existing channel in the extracellular half when the tilt is more developed. Overall, the photoinduced 13-*cis* polyene chain acts as the gate, and its tilt opens the channel that detours the constriction site.

The newly proposed, to our knowledge, pathway of the photoactivated ion-conduction alternative to the one through the opened constriction site explains the noncritical behavior of the mutation of the glutamate at the constriction site with nontitratable residues well (12,16) because the pathway detours the constriction site. Furthermore, remarkable activity changes by mutations at the DC-gate (39) can

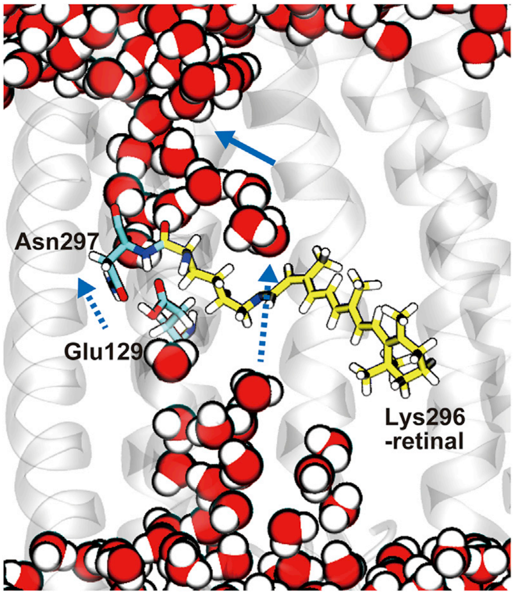

FIGURE 6 Ion-conduction pathways proposed in the eP_2 structure. A snapshot structure of an equilibrium MD trajectory of the eP2 state is shown. A newly proposed pathway goes by the 13-*cis* retinal chromophore and thus is different from a previously proposed pathway through the constriction site composed of Asn297 and Glu129, which is suggested to open upon deprotonation of Glu129. To see this figure in color, go online.

be well understood by the gating mechanism of our model. Mutagenesis experiments showed that replacement of Cys165 with threonine and that of its hydrogen-bond partner, Asp195, with alanine greatly increase the lifetime of the open state and thus are utilized to induce prolonged depolarization of neurons in optogenetics (59,60). The behavior of the mutants at the DC gate is hardly explained by the gating at the constriction site proposed previously, as the DC gate is located far from the constriction site (Fig. 2 *a*). On the other hand, in this model of the eP_2 state, the 13-methyl group of the chromophore in the tilted conformation strongly interacts with the side chain of Cys165, and therefore the mutations at the DC gate are naturally expected to greatly affect the stability of the tilted conformation of the chromophore leading to the open state.

The tilting conformational change of the chromophore in the photoactivation of ChR is different from known conformational changes in rhodopsins of archaea such as bR and sensory rhodopsin. In the photoactivation process of bR, which functions as a light-driven active proton transporter, the 13-methyl group in the planar conformation, without tilting, pushes a tryptophan residue in helix 6 located on the cytoplasmic side and in turn induces significant outward movements of helix 6 that fulfill the alternating access of transport (6). Sensory rhodopsin shares the common mechanism to generate signal transduction to its transducer through the outward movement of helix 6 (61). In the case of the active transport function of bR, the RPSB chromophore acts as a switch that temporally regulates the accessibility to the channels in the cytoplasmic half and the extracellular one in the photoactivation process. The *cis-trans* conformational change restricted in the planar form in the tight binding pocket is therefore necessary to strictly switch the accessibility to avoid adverse transport. On the other hand, the passive transport function of ChR requires the temporal connection between the cytoplasmic channel and the extracellular one, and thus the binding pocket is loosened to permit the tilting conformational changes of the chromophore to create the pathway of the ion conduction (Fig. 3).

Recently, Ardevol and Hummer proposed a mechanism of the channel formation of ChR2 different from the previous and these ones through different molecular simulation approaches (19). They employed a metadynamics technique to simulate the formation of the first photointermediate state, P_1, from the dark state and observed that PSB of the chromophore forms a salt bridge with Asp253 (Asp292 in C1C2) (Fig. 2 *a*), which was not seen in our simulation. The salt-bridge formation then correlated with change of a hydrogen-bond partner of Glu90 (Asp129 in C1C2) at the constriction site from Asn258 (Asn297 in C1C2) to Glu123 (Glu162 in C1C2), which was a counterion of PSB in the dark state (Fig. 2 *a*), and the dissociation of the hydrogen bond between Glu90 and Asn258 led to a water-pore formation connecting the channels in the cytoplasmic and extracellular halves already in the P_1 state. The water pore was therefore formed in the same region as that proposed previously by Gerwert and co-workers (12,13) (Fig. 6), albeit Glu90 was not deprotonated. In the present simulation, on the other hand, the hydrogen bond between Glu129 and Asn297 was kept intact and a water-pore formation at the constriction site was not observed.

Because the simulation study by Ardevol and Hummer was performed for a protein structural model of ChR2 constructed based on the x-ray crystallographic structure of the chimeric protein C1C2 (4), the mechanism can be different from that of this study obtained by the simulations for C1C2 itself. Nevertheless, several experimental observations do not seem to be explained by the mechanism proposed by Ardevol and Hummer well. The FTIR measurements (14,16) showed that upon the formation of the photoactivated state, vibrational frequency of the C=O stretching mode of Glu129 in C1C2 did not undergo any shift and that of Glu90 in ChR2 exhibited slight upshift, respectively. Those behaviors of frequency shift indicate that the strength of the hydrogen-bond of Glu129 in C1C2 does not change and that of Glu90 in ChR2 slightly decreases; the weakened hydrogen bond increases the double-bond character of the C=O bond in the resonance structure of the carboxyl group. However, the change of the hydrogen-bond partner of Glu90 from the neutral group of Asp258 to the anionic one of Glu123 proposed by Ardevol and Hummer is expected to largely strengthen the hydrogen-bond of Glu90 and thus is not in line with the experimental observations. Furthermore, the water-pore formation already in the P_1 state possibly introduces a proton conduction before the P_2 state, which was not seen in the electrophysiology observation. On the other hand, the hydrogen bond of Glu129 with Asp297 maintaining the constriction site during the photoactivation process in the present simulations explains the experimental evidence well.

The metadynamics technique used in the simulation by Ardevol and Hummer may not be a better one to model the first photointermediate state, P_1. To properly initiate the photocycle during which the functional conformational changes are induced, the conformational state of P_1 needs to be energetically relatively high and to be separated from the dark state by a high free-energy barrier, avoiding unfavorable deactivation to the dark state. In the formation of the first intermediate state through the photoisomerization reaction, the conformational trajectory easily leaps over the high free-energy barrier through a path on the energy surface of the electronically excited state on a timescale of less than a picosecond (28,62). However, the metadynamics simulation with a trajectory for a much longer time than the timescale of the excited-state process searches a low-energy state by crossing the lowest barrier by design and thus does not theoretically guarantee that the high-energy first photointermediate state is found. On the other hand, the procedure in the present study mimicked the photoexcited state process well (57,58)

and in fact successfully simulated the conformational changes of bR in the formations of the early intermediate states in a previous study (24), which was experimentally confirmed recently (30).

Because these results were obtained for the chimeric ChR, C1C2, the mechanism can be different from that of ChR2. However, the mechanism proposed in this study also successfully explains much of the experimental evidence that the mechanisms previously proposed hardly do, as described above. It will therefore be worth considering this mechanism for ChR2 as well in future studies.

FTIR and electrophysiology measurements showed that the channel opening is not established in the eP_2 formation and develops on a submillisecond timescale after the eP_2 formation, accompanied by more extensive hydration of α-helixes (15). A complete channel connection is not established yet in this atomistic model of the eP_2 state as observed experimentally, although the model uncovered a to our knowledge novel pathway of the ion conduction. A modeling of the open state is now ongoing with the QM/MM RWFE-SCF method, which enables highly accurate structural and energetic examinations and will provide the atomistic basis for improvement of the channel properties through rational design.

SUPPORTING MATERIAL

Seven figures and two tables are available at http://www.biophysj.org/biophysj/supplemental/S0006-3495(18)30978-0.

AUTHOR CONTRIBUTIONS

C.C., M.K., N.Y., and S.H. conceived the idea and designed the calculations. C.C. performed the molecular simulations. N.Y. performed the statistical analysis with the integral equation theory. M.K. performed the preliminary molecular simulations. M.T., R.I., and O.N. constructed the initial MD simulation system. C.C., N.Y., and S.H. wrote the manuscript. All authors contributed to the discussion of the study.

ACKNOWLEDGMENTS

We thank Tomohiro Nishizawa for fruitful discussions and comments.

This work was financially supported by grants from the Japanese Ministry of Education, Culture, Sports, Science and Technology (25104004, 16H04776, 18H05161, 16H00842, 16K05519, and "Priority Issue on Post-Kcomputer" (Building Innovative Drug Discovery Infrastructure Through Functional Control of Biomolecular Systems)). Computations were partly performed at the Research Institute for Information Technology (Kyushu University) and the Research Center for Computational Science, Okazaki, Japan.

REFERENCES

1. Deisseroth, K. 2011. Optogenetics. *Nat. Methods.* 8:26–29.
2. Tye, K. M., and K. Deisseroth. 2012. Optogenetic investigation of neural circuits underlying brain disease in animal models. *Nat. Rev. Neurosci.* 13:251–266.
3. Scholl, H. P., R. W. Strauss, ..., J. A. Sahel. 2016. Emerging therapies for inherited retinal degeneration. *Sci. Transl. Med.* 8:368rv6.
4. Kato, H. E., F. Zhang, ..., O. Nureki. 2012. Crystal structure of the channelrhodopsin light-gated cation channel. *Nature.* 482:369–374.
5. Volkov, O., K. Kovalev, ..., V. Gordeliy. 2017. Structural insights into ion conduction by channelrhodopsin 2. *Science.* 358:eaan8862.
6. Ernst, O. P., D. T. Lodowski, ..., H. Kandori. 2014. Microbial and animal rhodopsins: structures, functions, and molecular mechanisms. *Chem. Rev.* 114:126–163.
7. Grote, M., M. Engelhard, and P. Hegemann. 2014. Of ion pumps, sensors and channels - perspectives on microbial rhodopsins between science and history. *Biochim. Biophys. Acta.* 1837:533–545.
8. Lórenz-Fonfría, V. A., and J. Heberle. 2014. Channelrhodopsin unchained: structure and mechanism of a light-gated cation channel. *Biochim. Biophys. Acta.* 1837:626–642.
9. Nagel, G., D. Ollig, ..., P. Hegemann. 2002. Channelrhodopsin-1: a light-gated proton channel in green algae. *Science.* 296:2395–2398.
10. Nagel, G., T. Szellas, ..., E. Bamberg. 2003. Channelrhodopsin-2, a directly light-gated cation-selective membrane channel. *Proc. Natl. Acad. Sci. USA.* 100:13940–13945.
11. Feldbauer, K., D. Zimmermann, ..., E. Bamberg. 2009. Channelrhodopsin-2 is a leaky proton pump. *Proc. Natl. Acad. Sci. USA.* 106:12317–12322.
12. Eisenhauer, K., J. Kuhne, ..., K. Gerwert. 2012. In channelrhodopsin-2 Glu-90 is crucial for ion selectivity and is deprotonated during the photocycle. *J. Biol. Chem.* 287:6904–6911.
13. Kuhne, J., K. Eisenhauer, ..., F. Bartl. 2015. Early formation of the ion-conducting pore in channelrhodopsin-2. *Angew. Chem. Int.Engl.* 54:4953–4957.
14. Lórenz-Fonfría, V. A., T. Resler, ..., J. Heberle. 2013. Transient protonation changes in channelrhodopsin-2 and their relevance to channel gating. *Proc. Natl. Acad. Sci. USA.* 110:E1273–E1281.
15. Lórenz-Fonfría, V. A., C. Bamann, ..., J. Heberle. 2015. Temporal evolution of helix hydration in a light-gated ion channel correlates with ion conductance. *Proc. Natl. Acad. Sci. USA.* 112:E5796–E5804.
16. Inaguma, A., H. Tsukamoto, ..., Y. Furutani. 2015. Chimeras of channelrhodopsin-1 and -2 from Chlamydomonas reinhardtii exhibit distinctive light-induced structural changes from channelrhodopsin-2. *J. Biol. Chem.* 290:11623–11634.
17. Watanabe, H. C., K. Welke, ..., M. Elstner. 2013. Towards an understanding of channelrhodopsin function: simulations lead to novel insights of the channel mechanism. *J. Mol. Biol.* 425:1795–1814.
18. Takemoto, M., H. E. Kato, ..., O. Nureki. 2015. Molecular dynamics of channelrhodopsin at the early stages of channel opening. *PLoS One.* 10:e0131094.
19. Ardevol, A., and G. Hummer. 2018. Retinal isomerization and water-pore formation in channelrhodopsin-2. *Proc. Natl. Acad. Sci. USA.* 115:3557–3562.
20. Müller, M., C. Bamann, ..., W. Kühlbrandt. 2015. Light-induced helix movements in channelrhodopsin-2. *J. Mol. Biol.* 427:341–349.
21. Krause, N., C. Engelhard, ..., R. Bittl. 2013. Structural differences between the closed and open states of channelrhodopsin-2 as observed by EPR spectroscopy. *FEBS Lett.* 587:3309–3313.
22. Sattig, T., C. Rickert, ..., C. Bamann. 2013. Light-induced movement of the transmembrane helix B in channelrhodopsin-2. *Angew. Chem. Int.Engl.* 52:9705–9708.
23. Hayashi, S., and I. Ohmine. 2000. Proton transfer in bacteriorhodopsin: structure, excitation, IR spectra, and potential energy surface analyses by an ab initio QM/MM method. *J. Phys. Chem. B.* 104:10678–10691.
24. Hayashi, S., E. Tajkhorshid, and K. Schulten. 2002. Structural changes during the formation of early intermediates in the bacteriorhodopsin photocycle. *Biophys. J.* 83:1281–1297.
25. Bondar, A. N., S. Fischer, ..., J. C. Smith. 2005. Tuning of retinal twisting in bacteriorhodopsin controls the directionality of the early photocycle steps. *J. Phys. Chem. B.* 109:14786–14788.

26. Dokukina, I., and O. Weingart. 2015. Spectral properties and isomerisation path of retinal in C1C2 channelrhodopsin. *Phys. Chem. Chem. Phys.* 17:25142–25150.

27. VanGordon, M. R., G. Gyawali, …, S. B. Rempe. 2017. Atomistic study of intramolecular interactions in the closed-state channelrhodopsin chimera, C1C2. *Biophys. J.* 112:943–952.

28. Hontani, Y., M. Marazzi, …, J. T. M. Kennis. 2017. Reaction dynamics of the chimeric channelrhodopsin C1C2. *Sci. Rep.* 7:7217.

29. Adam, S., and A. N. Bondar. 2018. Mechanism by which water and protein electrostatic interactions control proton transfer at the active site of channelrhodopsin. *PLoS One.* 13:e0201298.

30. Nango, E., A. Royant, …, S. Iwata. 2016. A three-dimensional movie of structural changes in bacteriorhodopsin. *Science.* 354:1552–1557.

31. Kosugi, T., and S. Hayashi. 2012. QM/MM reweighting free energy SCF for geometry optimization on extensive free energy surface of enzymatic reaction. *J. Chem. Theory Comput.* 8:322–334.

32. Hayashi, S., Y. Uchida, …, M. Kamiya. 2017. QM/MM geometry optimization on extensive free-Energy surfaces for examination of enzymatic reactions and design of novel functional properties of proteins. *Annu. Rev. Phys. Chem.* 68:135–154.

33. Kamiya, M., and S. Hayashi. 2017. Photoactivation intermediates of a G-protein coupled receptor rhodopsin investigated by a hybrid molecular simulation. *J. Phys. Chem. B.* 121:3842–3852.

34. Kato, H. E., M. Kamiya, …, O. Nureki. 2015. Atomistic design of microbial opsin-based blue-shifted optogenetics tools. *Nat. Commun.* 6:7177.

35. Beglov, D., and B. Roux. 1996. Solvation of complex molecules in a polar liquid: an integral equation theory. *J. Chem. Phys.* 104:8678–8689.

36. Beglov, D., and B. Roux. 1997. An integral equation to describe the solvation of polar molecules in liquid water. *J. Phys. Chem. B.* 101:7821–7826.

37. Kovalenko, A., and F. Hirata. 1998. Three-dimensional density profiles of water in contact with a solute of arbitrary shape: a RISM approach. *Chem. Phys. Lett.* 290:237–244.

38. Kovalenko, A., and F. Hirata. 1999. Self-consistent description of a metal-water interface by the Kohn-Sham density functional theory and the three-dimensional reference interaction site model. *J. Chem. Phys.* 110:10095–10112.

39. Nack, M., I. Radu, …, J. Heberle. 2010. The DC gate in Channelrhodopsin-2: crucial hydrogen bonding interaction between C128 and D156. *Photochem. Photobiol. Sci.* 9:194–198.

40. Olsson, M. H., C. R. Søndergaard, …, J. H. Jensen. 2011. PROPKA3: consistent treatment of internal and surface residues in empirical pKa predictions. *J. Chem. Theory Comput.* 7:525–537.

41. Kosugi, T., and S. Hayashi. 2012. Crucial role of protein flexibility in formation of a stable reaction transition state in an α-amylase catalysis. *J. Am. Chem. Soc.* 134:7045–7055.

42. Phillips, J. C., R. Braun, …, K. Schulten. 2005. Scalable molecular dynamics with NAMD. *J. Comput. Chem.* 26:1781–1802.

43. Schmidt, M. W., K. K. Baldridge, …, J. A. Montgomery. 1993. General atomic and molecular electronic structure system. *J. Comput. Chem.* 14:1347–1363.

44. MacKerell, A. D., Jr., N. Banavali, and N. Foloppe. 2000–2001. Development and current status of the CHARMM force field for nucleic acids. *Biopolymers.* 56:257–265.

45. Huang, J., and A. D. MacKerell, Jr. 2013. CHARMM36 all-atom additive protein force field: validation based on comparison to NMR data. *J. Comput. Chem.* 34:2135–2145.

46. Kamiya, M., H. E. Kato, …, S. Hayashi. 2013. Structural and spectral characterizations of C1C2 channelrhodopsin and its mutants by molecular simulations. *Chem. Phys. Lett.* 556:266–271.

47. Jorgensen, W. L., J. Chandrasekhar, …, M. L. Klein. 1983. Comparison of simple potential functions for simulating liquid water. *J. Chem. Phys.* 79:926–935.

48. Steinbach, P. J., and B. R. Brooks. 1994. New spherical-cutoff methods for long-range forces in macromolecular simulation. *J. Comput. Chem.* 15:667–683.

49. Essmann, U., L. Perera, …, L. G. Pedersen. 1995. A smooth particle mesh Ewald method. *J. Chem. Phys.* 103:8577–8593.

50. Maruyama, Y., and F. Hirata. 2012. Modified anderson method for accelerating 3D-RISM calculations using graphics processing unit. *J. Chem. Theory Comput.* 8:3015–3021.

51. Maruyama, Y., N. Yoshida, …, F. Hirata. 2014. Massively parallel implementation of 3D-RISM calculation with volumetric 3D-FFT. *J. Comput. Chem.* 35:1347–1355.

52. Humphrey, W., A. Dalke, and K. Schulten. 1996. VMD: visual molecular dynamics. *J. Mol. Graph.* 14:33–38, 27–28.

53. Ito, S., H. E. Kato, …, H. Kandori. 2014. Water-containing hydrogen-bonding network in the active center of channelrhodopsin. *J. Am. Chem. Soc.* 136:3475–3482.

54. Edman, K., P. Nollert, …, E. M. Landau. 1999. High-resolution X-ray structure of an early intermediate in the bacteriorhodopsin photocycle. *Nature.* 401:822–826.

55. Luecke, H., B. Schobert, …, J. K. Lanyi. 2000. Coupling photoisomerization of retinal to directional transport in bacteriorhodopsin. *J. Mol. Biol.* 300:1237–1255.

56. Luecke, H., B. Schobert, …, J. K. Lanyi. 1999. Structure of bacteriorhodopsin at 1.55 A resolution. *J. Mol. Biol.* 291:899–911.

57. Hayashi, S., E. Tajkhorshid, and K. Schulten. 2003. Molecular dynamics simulation of bacteriorhodopsin's photoisomerization using ab initio forces for the excited chromophore. *Biophys. J.* 85:1440–1449.

58. Hayashi, S., E. Tajkhorshid, and K. Schulten. 2009. Photochemical reaction dynamics of the primary event of vision studied by means of a hybrid molecular simulation. *Biophys. J.* 96:403–416.

59. Berndt, A., O. Yizhar, …, K. Deisseroth. 2009. Bi-stable neural state switches. *Nat. Neurosci.* 12:229–234.

60. Bamann, C., R. Gueta, …, E. Bamberg. 2010. Structural guidance of the photocycle of channelrhodopsin-2 by an interhelical hydrogen bond. *Biochemistry.* 49:267–278.

61. Moukhametzianov, R., J. P. Klare, …, V. I. Gordeliy. 2006. Development of the signal in sensory rhodopsin and its transfer to the cognate transducer. *Nature.* 440:115–119.

62. Verhoefen, M. K., C. Bamann, …, J. Wachtveitl. 2010. The photocycle of channelrhodopsin-2: ultrafast reaction dynamics and subsequent reaction steps. *ChemPhysChem.* 11:3113–3122.

Biophysical Journal, Volume *115*

Supplemental Information

An Atomistic Model of a Precursor State of Light-Induced Channel Opening of Channelrhodopsin

Cheng Cheng, Motoshi Kamiya, Mizuki Takemoto, Ryuichiro Ishitani, Osamu Nureki, Norio Yoshida, and Shigehiko Hayashi

Table S1 Dihedral angles of the polyene chain of the chromophore (degrees). Values in parenthesis are deviations from the planarity.

	X-Ray (3UG9)	D_0	P_1	eP_2	eP_2'
C_5=C_6	-0.9 (0.9)	7.1 (7.1)	7.4 (7.4)	12.1 (12.1)	7.5 (7.5)
C_6–C_7	177.7 (2.3)	173.7 (6.3)	173.0 (7.0)	-176.9 (3.1)	175.3 (4.7)
C_7=C_8	178.2 (1.8)	-178.0 (2.0)	-174.0 (6.0)	-169.2 (10.8)	-177.1 (2.9)
C_8–C_9	-173.0 (7.0)	-176.0 (4.0)	177.7 (2.3)	-179.0 (1.0)	-179.0 (1.0)
C_9=C_{10}	179.8 (0.2)	173.6 (6.4)	-173.8 (6.2)	-171.0 (9.0)	-177.9 (2.1)
C_{10}–C_{11}	-174.0 (6.0)	-172.0 (8.0)	-179.8 (0.2)	-170.0 (10.0)	-176.0 (4.0)
C_{11}=C_{12}	179.4 (0.6)	170.4 (9.6)	-169.3 (10.7)	-169.8 (10.2)	-174.2 (5.8)
C_{12}–C_{13}	172.3 (7.7)	-178.5 (1.5)	171.6 (8.4)	-179.1 (0.9)	172.3 (7.7)
C_{13}=C_{14}	-179.6 (0.4)	167.2 (12.8)	25.8 (25.8)	6.3 (6.3)	20.6 (20.6)
C_{14}–C_{15}	-174.3 (5.7)	176.9 (3.1)	-173.4 (6.6)	-160.5 (19.5)	-166.4 (13.6)
C_{15}=N_ζ	167.1 (12.9)	176.5 (3.5)	-154.9 (25.1)	-177.1 (2.9)	-161.9 (18.1)

Table S2 Comparison of energies between the eP_2 and eP_2' states.

	E_{QM}[a]	$V_{QM\text{-}MM}$[b]
eP_2	-912.4657	-0.1354
eP_2'	-912.4588	-0.1330
	ΔE_{QM}[c]	$\Delta V_{QM\text{-}MM}$[c]
eP_2 – eP_2'	-4.3	-1.5

[a] Expectation values of the QM Hamiltonian (Hartree).

[b] Mean QM-MM interaction energies obtained at the converged cycles of QM/MM RWFE-SCF optimizations (Hartree).

[c] Difference in the energies between the eP_2 and eP_2' states (kcal/mol).

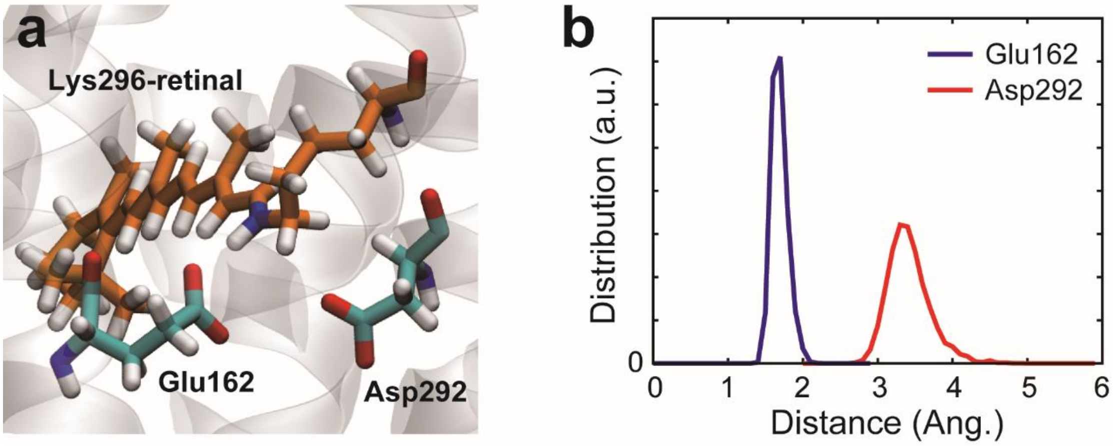

Figure S1 Salt-bridge formation of PSB with its counter ion carboxylate. a A snapshot structure around PSB from an equilibrium MD trajectory of the D_0 state. b Statistical distributions of distances between H_ζ atom of PSB and the closest oxygen atoms of carboxyl groups of its counter ion carboxylates, Glu162 and Asp292, in an equilibrium MD trajectory of the D_0 state.

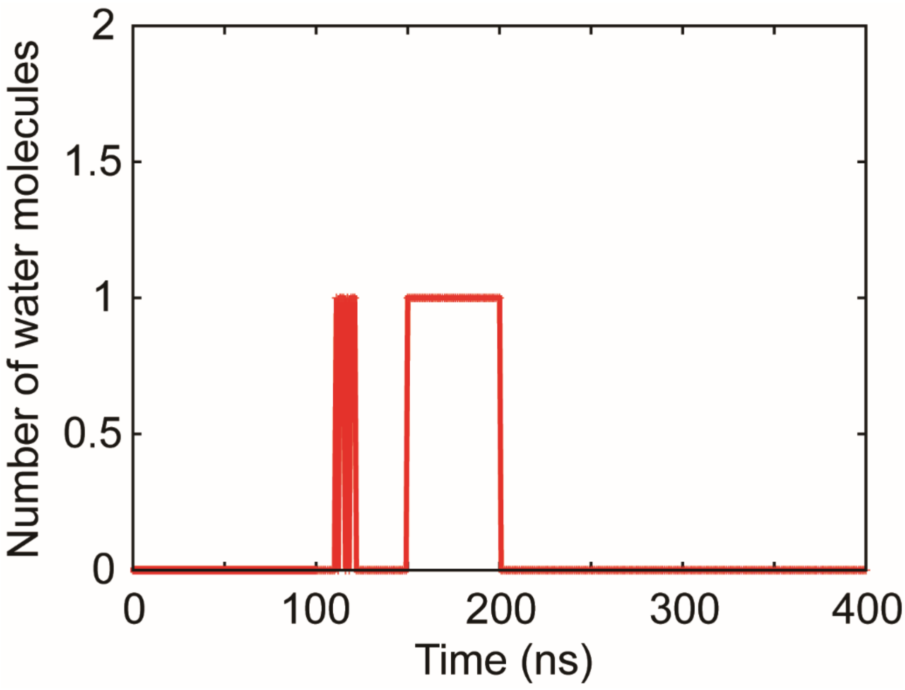

Figure S2 Transient occupation of a water molecule in a cavity at the DC-gate in an equilibrium MD simulation of the D_0 state. Water molecules of which the oxygen atoms are within 4.5 Å from the carbon atom of the carboxyl group of Asp195 were counted.

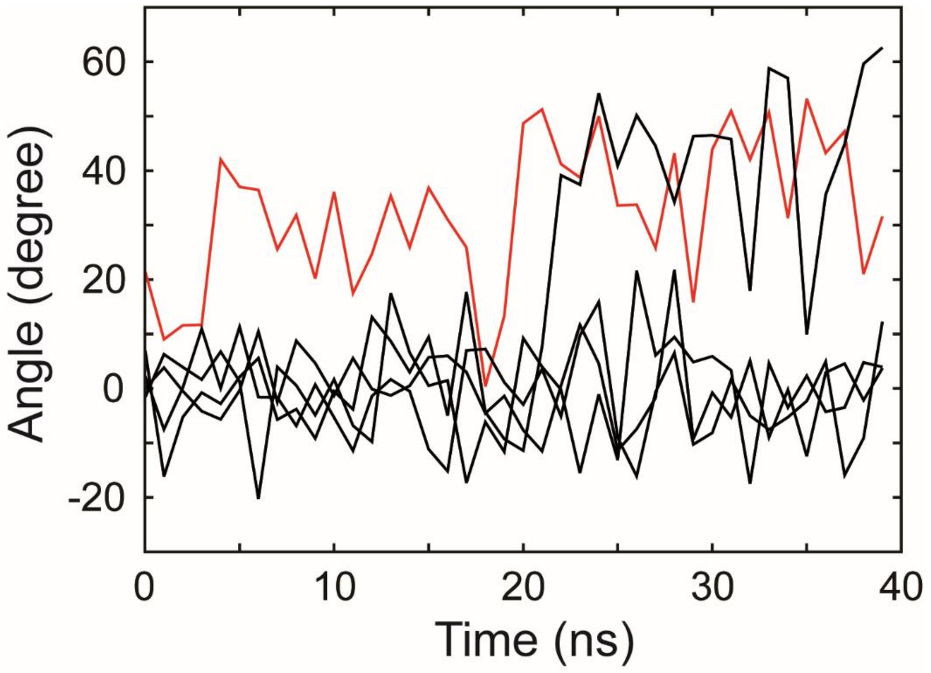

Figure S3 Time evolution of a dihedral angle of C(5-Me)-C_5-C_{13}-C(13-Me) of the chromophore during non-equilibrium MD simulations of the eP_2 state formation where C(5-Me) and C(13-Me) are the carbon atoms of methyl groups at the positions of 5 and 13, respectively. A red line indicates the time evolution of a trajectory which was followed by a QM/MM free energy geometry optimization shown in Figure S4.

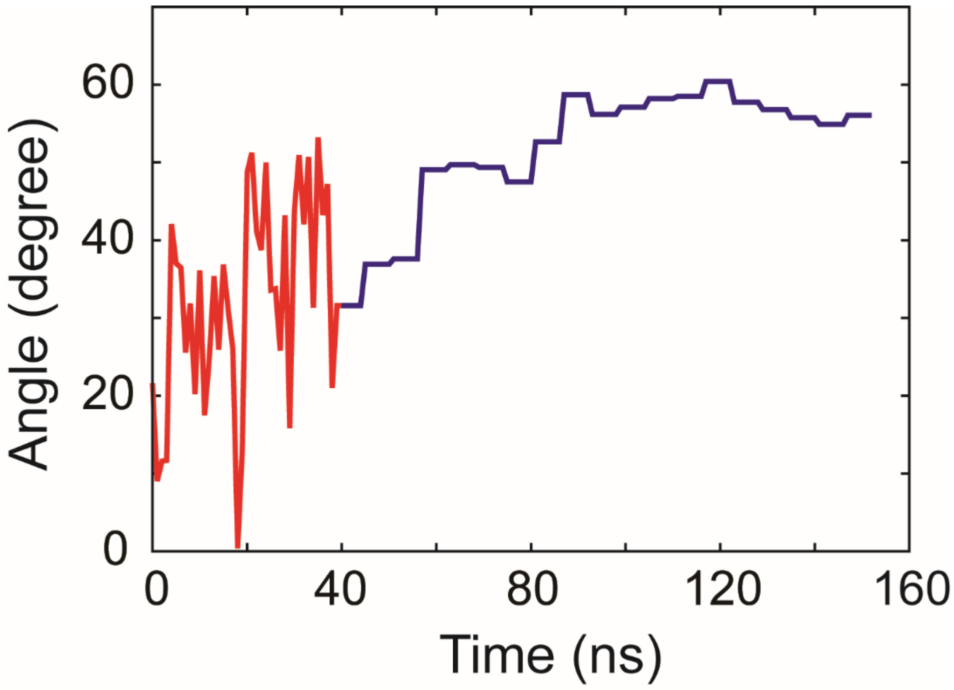

Figure S4 Time evolution of a dihedral angle of C(5-Me)-C_5-C_{13}-C(13-Me) of the chromophore during a non-equilibrium MD simulation (red) and a QM/MM free energy geometry optimization (blue) of formation of the eP_2 state.

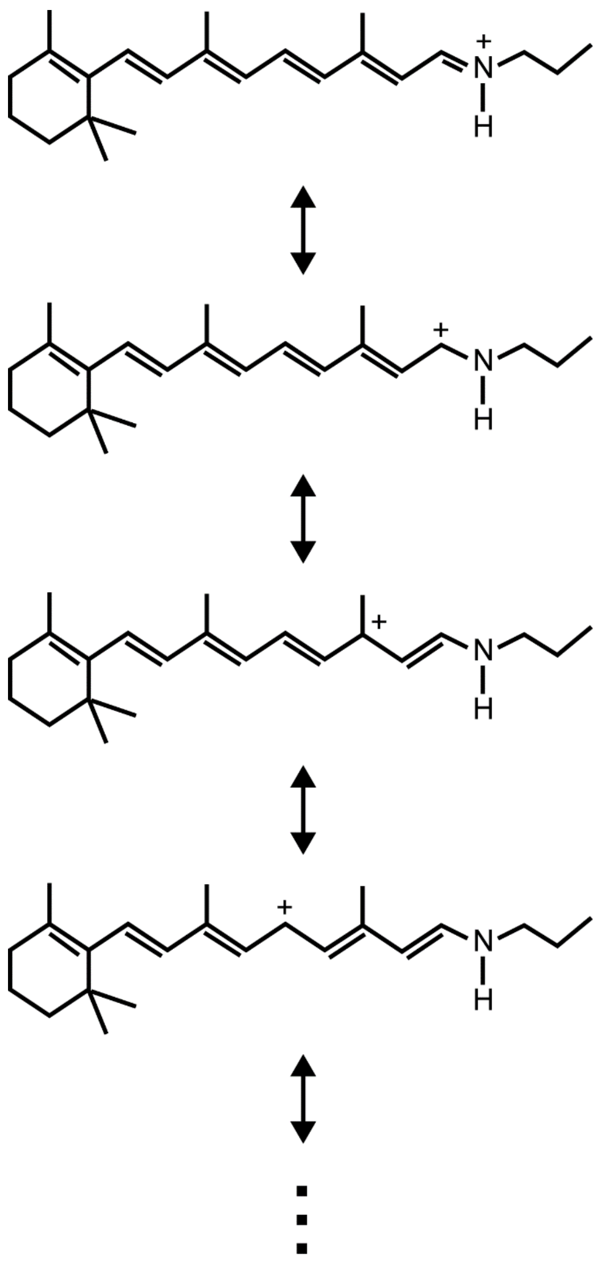

Figure S5 Resonance electronic structures of retinal protonated Schiff base. Single and double bond characters of the polyene chain is more strongly mixed through the resonance structures. Note that the resonance structures are very unstable in the deprotonated form of retinal Schiff base, and thus its single and double bond characters are more pronounced.

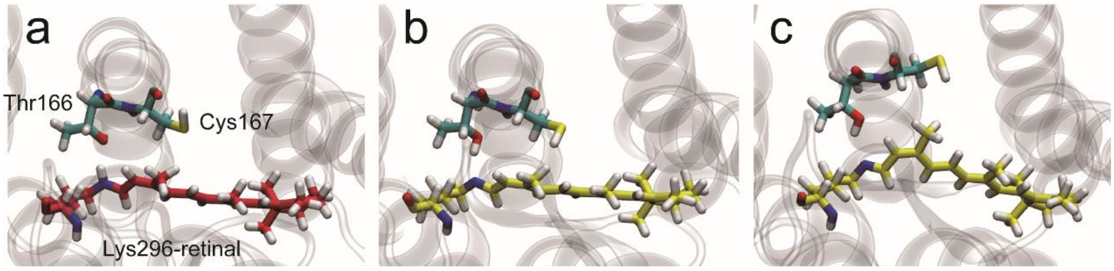

Figure S6 Free energetically minimum structures of the chromophore during formation of the eP_2 state. a-c Views of the chromophore binding site from the cytoplasmic side in the P_1 (a), eP_2' (b), and eP_2 (c) states. The structures of the P_1 and eP_2 states are the same as those shown in Figure 2c,e, respectively.

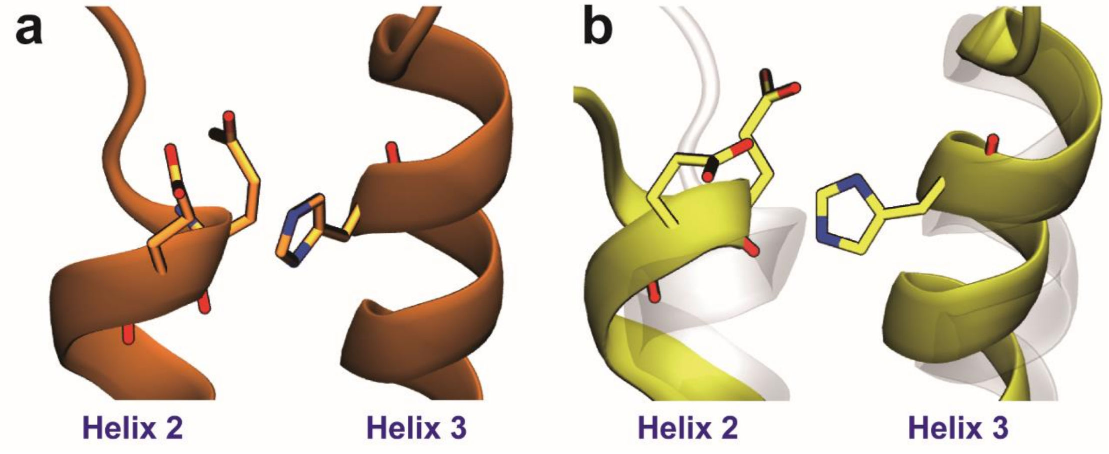

Figure S7 Conformational changes of cytoplasmic ends of Helixes 2 and 3 upon formation of the eP_2 state. a-b Snapshot structures of cytoplasmic ends of Helixes 2 and 3 in the D_0 state (a) and the eP_2 state (b), respectively, taken from equilibrium MD simulations. Side-chains of Glu121 and Glu122 in Helix 2 and that of His173 are depicted in licorice representation. The structure in the D_0 state is also drawn transparently in b.
